# [Retracted] Suppression of SIK1 by miR-141 in human ovarian cancer cell lines and tissues

**DOI:** 10.3892/ijmm.2025.5700

**Published:** 2025-11-24

**Authors:** Jin-Long Chen, Fang Chen, Ting-Ting Zhang, Nai-Fu Liu

Int J Mol Med 37: 1601-1610, 2016; DOI: 10.3892/ijmm.2016.2553

Following the publication of this paper, a concerned reader drew to the Editor's attention that, regarding the western blots shown in Fig. 1A, where the expression levels of salt-inducible kinase 1 were detected in ovarian cancer tissues, the β-actin loading controls for patients 4, 6 and 7 were strikingly similar in appearance [also note that, in view of having not received a timely response to this query from the authors, an Expression of concern (DOI: 10.3892/ijmm.2025.5627) was published for this paper]. Moreover, several of the protein bands in this figure were strikingly similar to data which subsequently appeared in another paper written by different authors at different research institutes that was also published in the journal *Experimental and Therapeutic Medicine*, although this paper has since been retracted.

The authors have subsequently replied to say that they are unable to retrieve a portion of the original experimental data, and therefore wish to retract this paper. All the authors agree with the retraction of this article. Both the authors and the Editor of *International Journal of Molecular Medicine* apologize to the readership for any inconvenience caused.

